# Dermatological manifestations and its association with SARS-CoV-2: a descriptive cross-sectional study from Guatemala^☆^^☆☆^

**DOI:** 10.1016/j.abd.2020.08.005

**Published:** 2020-11-17

**Authors:** Azucena Hernández Rousselin

**Affiliations:** Department of Internal Medicine, Dermatology Unit, Hospital Roosevelt, Guatemala, Guatemala

*Dear Editor,*

SARS-CoV-2 has a wide spectrum of symptoms, ranging from mild cold-like illness, severe respiratory distress, multi-system disease, and death. There are a few reports regarding cutaneous manifestations; reported findings were: rash, urticaria, chickenpox-like vesicles, livedo reticularis, herpetiform lesions, and chilblains.^1^, ^2^ A descriptive cross-sectional study was carried out at Roosevelt Hospital of Guatemala, a first-level infirmary located in the capital city. The sample was determined based on the reported prevalence, which ranges from 1.5%, 7.8% to 20%.^1^, ^3^, ^4^ An expected percentage of 9% was estimated, with a sampling error of 4% and a confidence level of 95%. A total of 202 patients were included, 77 women (38.1) and 125 (61.9%) men. The age ranged from 12 to 82 years, with a mean of 48.9. The most frequent comorbidities were diabetes (63; 31.2%), hypertension (41; 20.3%), chronic kidney disease (nine; 4.5%), and obesity (14; 6.9%). It was found that 12 patients had skin manifestations, of which five (2.5%) were directly associated with SARS-CoV2 (Figure 1, Figure 2, Figure 3), and seven (3.5%) a circumstantial relationship with it. In the group of patients with direct association, there was no other explanation for the dermatological injury. One patient had reactive arthritis in the knee (this patient had sterile synovial fluid culture and no other associated infection), one patient had urticariform reaction, one patient had erythematous rash (they only received acetaminophen), and two patients had livedo reticular in the legs. The circumstantial association is based on the fact that there are injuries related to the treatment or immunological state of the patient. Three patients who were in prone position to improve oxygenation presented vascular macules on their knees; a biopsy was taken to rule out vasculitis, and only extravasated erythrocytes were found. Two patients presented monomorphic papules on the chest and forehead, but were receiving methylprednisolone, and the condition was diagnosed as steroid acne. Two immunosuppressed patients (HIV-positive and chronic kidney disease) presented vesicles on an erythematous base; the distribution in one case was dermatomal and in the other the lesions were grouped. These findings are clinically consistent with the Herpesviridae family viruses. In the literature, vesicular lesions similar to varicella are reported, with a more dispersed and diffuse distribution and located on the trunk.^3^, ^5^ Thus, the clinical and evolution characteristics can help to differentiate this particular injury. A polymerase chain reaction test of a sample of the lesion is very useful in the identification of the causative virus.^4^

**Figure 1:**
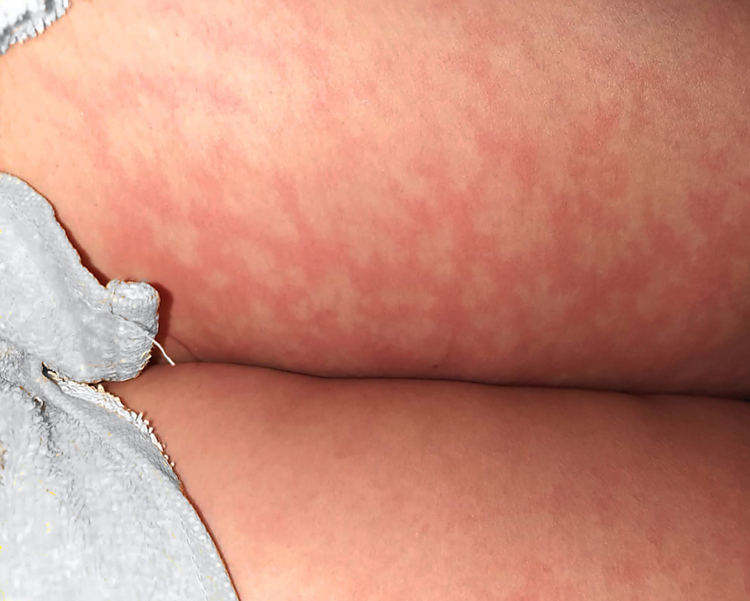
Livedo reticular.

**Figure 2:**
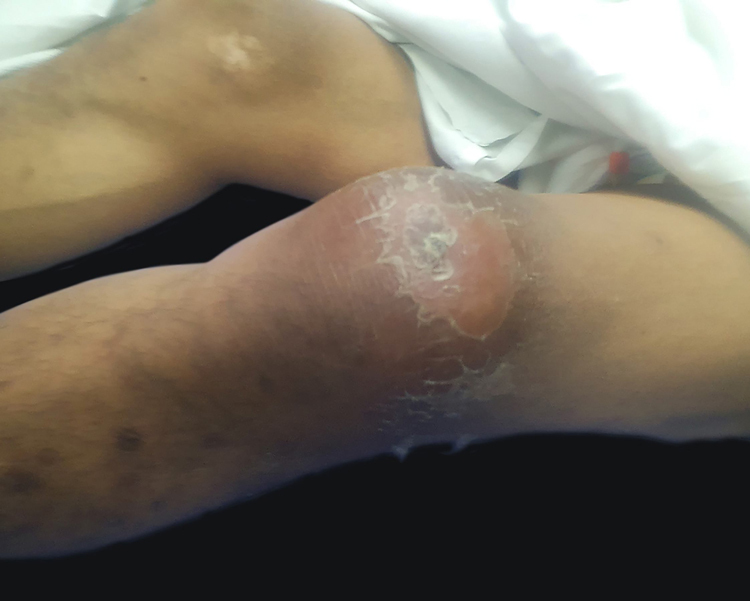
Reactive arthritis.

**Figure 3:**
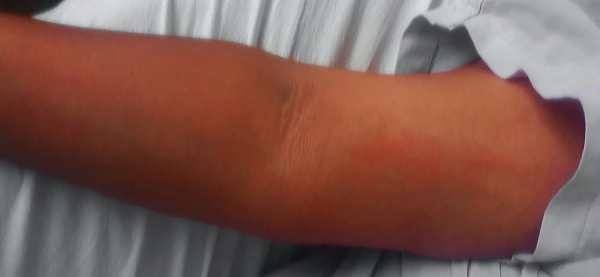
Urticariform reaction.

The prevalence of skin lesions observed in the present study was much lower than that found by Recalcati, but similar to that reported by Tammaro.^1^, ^3^

The skin manifestations found in this study are similar to those caused by other viruses, and it cannot be concluded that there is a pathognomonic skin lesion of SARS-CoV-2.

As previously reported, no correlation with disease severity was observed.^1^, ^4^ The deficit in the immune system can cause other infections, and the established therapy can also cause skin lesions; therefore, it is essential to carry out detailed studies in each case to make a better differential diagnosis.

## Financial support

None declared.

## Author contribution

Azucena Hernández Rousselin: Approval of the final version; study conception and planning; writing of the manuscript; data collection, analysis and interpretation.

## Conflicts of interest

None declared.
